# Orbital Langerhans Cell Histiocytosis: A Systematic Review of 228 Cases

**DOI:** 10.7759/cureus.71059

**Published:** 2024-10-08

**Authors:** Ahmed S Al-Wassiti, Ali A Bani-Saad, Mohammed A Bani Saad, Mustafa Ismail

**Affiliations:** 1 Department of Surgery, College of Medicine, University of Baghdad, Baghdad, IRQ; 2 Department of Surgery, Baghdad Teaching Hospital, Medical City Complex, Baghdad, IRQ

**Keywords:** langerhans cell histiocytosis, multidisciplinary management, ophthalmology education, orbital decompression surgery, orbital histiocytosis

## Abstract

Orbital Langerhans cell histiocytosis (LCH) is an extremely rare disorder, and widely different manifestations often make it diagnostically challenging. The variability of symptoms - from common presentations, such as eyelid swelling and exophthalmos, to very atypical symptoms, like headaches and diplopia - frequently results in delayed diagnosis and mismanagement. This systematic review aims to describe in detail the clinical presentation, diagnostic approaches, treatment modalities, and outcomes of orbital LCH. This systematic review was performed based on the Preferred Reporting Items for Systematic Reviews and Meta-Analyses (PRISMA) guidelines. Literature searches were conducted in PubMed and Scopus up to August 2024. Synthesis of data involved 228 patients from 18 studies. Extracted data included study design, sample size, patient demographics (age, gender, ethnicity), clinical presentation, diagnostic criteria, treatment modalities, follow-up duration, recurrence rates, and complications.

The review found that orbital LCH mostly affects subjects with a mean age of 8.5 years (SD ± 7.1 years), indicating that a greater number of subjects are from younger populations, with 152 males (66.7%) and 76 females (33.3%). The most common presenting symptom was eyelid swelling, reported in 108 patients (47.4%), often serving as the initial sign prompting further investigation, exophthalmos was observed in 95 patients (41.7%), indicating more significant orbital involvement, and palpable mass was detected in 80 patients (35.1%). Imaging played a critical role in the diagnosis, with CT or MRI revealing lytic lesions in nearly all cases (99%). Histopathology confirmed LCH with CD1a and S-100 proteins serving as hallmark markers of LCH. Treatment strategies for orbital LCH varied depending on the extent of disease, patient characteristics, and institutional practices. Surgical intervention was the most common treatment modality, used in 136 patients (59.6%), and it was very effective in localized disease. Radiation therapy was employed in 68 patients (29.8%), often as an adjunct to surgery or as a primary treatment for residual or inoperable disease. Chemotherapy was administered to 85 patients (37.3%), especially those with multisystem involvement. Observation and follow-up were employed in selected cases, particularly those with solitary or asymptomatic lesions, allowing for the possibility of spontaneous regression. Although the remission rate was high, at 79.8%, there was a recurrence in 14.9% of the patients, thus requiring close follow-up. Diabetes insipidus was a complication, and it was also a pointer to multisystem involvement. Orbital LCH is a diagnosis that requires a multidisciplinary approach for accurate diagnosis and effective management, with individualized treatment guided by advanced imaging and molecular markers. Further studies are needed to refine treatment protocols in an attempt to reduce recurrence rates.

## Introduction and background

Langerhans cell histiocytosis (LCH) is a very rare and enigmatic disorder exemplified by the clonal proliferation of Langerhans cells, which are specialized antigen-presenting dendritic cells. Normally, these cells take up and present antigens to T-cells in order to trigger immunological responses. On the other hand, LCH consists of abnormal proliferation of such cells with possible invasion into tissues and organs of almost all kinds, leading to a very wide spectrum of clinical manifestations. While virtually any organ system may become involved with the disease process, the most common sites of involvement include bones, skin, lungs, and the pituitary gland. Orbital involvement is relatively rare but of special significance anatomically and functionally [1].

LCH predominately causes illness in children. It mainly affects children from one to three years of age. Its overall incidence is estimated at four to five cases per million children annually. Still, there is a lack of proper recording, so the exact frequency of orbital LCH cannot be presented since it is a rare disorder. Orbital involvement is estimated to occur in about 20% of patients with LCH, either as part of multifocal disease or as an isolated unifocal lesion [2]. A slight male predominance was noted with a male-to-female ratio of about 2:1. Interestingly, while LCH is more common during childhood, it may also be present in adults, in whom it usually has a more indolent course.

Although the pathogenesis of LCH remains incompletely understood, it is thought to involve genetic mutations and immune dysregulation. Recent investigations into this disorder have unraveled somatic mutations in genes encoding elements of the mitogen-activated protein kinase (MAPK) pathway, especially the BRAF gene, which is present in a significant number of cases of LCH. It has also been hypothesized that environmental factors like infections or toxins have a role, but the evidence so far is not clear on this aspect [3].

Orbital LCH is almost always non-specifically manifested as proptosis, swelling of the eyelids, pain, and erythema, imitating other more common orbital disorders like cellulitis, orbital pseudotumor, or rhabdomyosarcoma. The involvement of orbital bones by osteolytic lesions, especially in the frontozygomatic bones, is characteristic and well picked up with imaging studies like CT or MRI. Histopathologically, diagnosis will be confirmed with the presence of Langerhans cells bearing markers such as CD1a and S100. The differential diagnosis for orbital LCH is very wide, including infectious, inflammatory, and neoplastic processes [4].

Diagnosis of orbital LCH is challenging due to it being a very rare entity with nonspecific symptoms. Early and accurate diagnosis in this series is crucial for the visual outcome and prognosis of the patient. There needs to be a high index of suspicion, especially in pediatric patients who have proptosis associated with osteolytic lesions. The gold standard remains lesion biopsy with subsequent histopathological examination and immunohistochemical staining. Imaging studies are now central to making the diagnosis but are also often used to define the extent of disease involvement and plan treatment [5].

The management of orbital LCH depends on the extent of disease involvement and the presence of systemic symptoms. This means that in isolated unifocal lesions, surgical excision may be curative, and additional treatment may not be necessary. In multifocal or multisystem diseases, systemic therapy with chemotherapy agents like vinblastine, associated with corticosteroids, is frequently necessary. Radiation therapy may also be used in incomplete resections or inoperable cases, although it is not frequently utilized because of probable long-term side effects. The decision to use systemic treatment is generally advised in the presence of high-risk features - successively detailed as the involvement of critical viscera, such as the liver, spleen, or central nervous system [6].

Even after progress has been made in understanding LCH, orbital LCH still remains a difficult disease to manage. The rarity of the disease limits the availability of large-scale clinical trials, and until now, no standardized treatment protocol has been institutionalized. Moreover, possible long-term complications, such as diabetes insipidus and neurogenerative changes, require close follow-up and multi-disciplinary care. Newer approaches targeting the MAPK pathway are emerging, and these treatments are very promising, but further studies need to be conducted to confirm their efficacy and safety in this patient population [7].

Given the heterogeneity and rarity of orbital LCH, there is a need for a comprehensive synthesis of the existing literature. This systematic review is intended to synthesize data from several studies on clinical manifestations, results of treatment, and prognostic factors accruing in orbital LCH.

A graphical abstract of this review is presented in the Appendices.

## Review

Methodology

Study Design

This systematic review was performed based on the Preferred Reporting Items for Systematic Reviews and Meta-Analyses (PRISMA) guidelines [8]. This systematic review was primarily done to synthesize all available literature related to LCH to present an overview of clinical features, treatment outcomes, and prognostic factors. Since it is an observational study, there is no need for ethical approval or formal written informed consent in this study.

Literature Search

**Figure 1:**
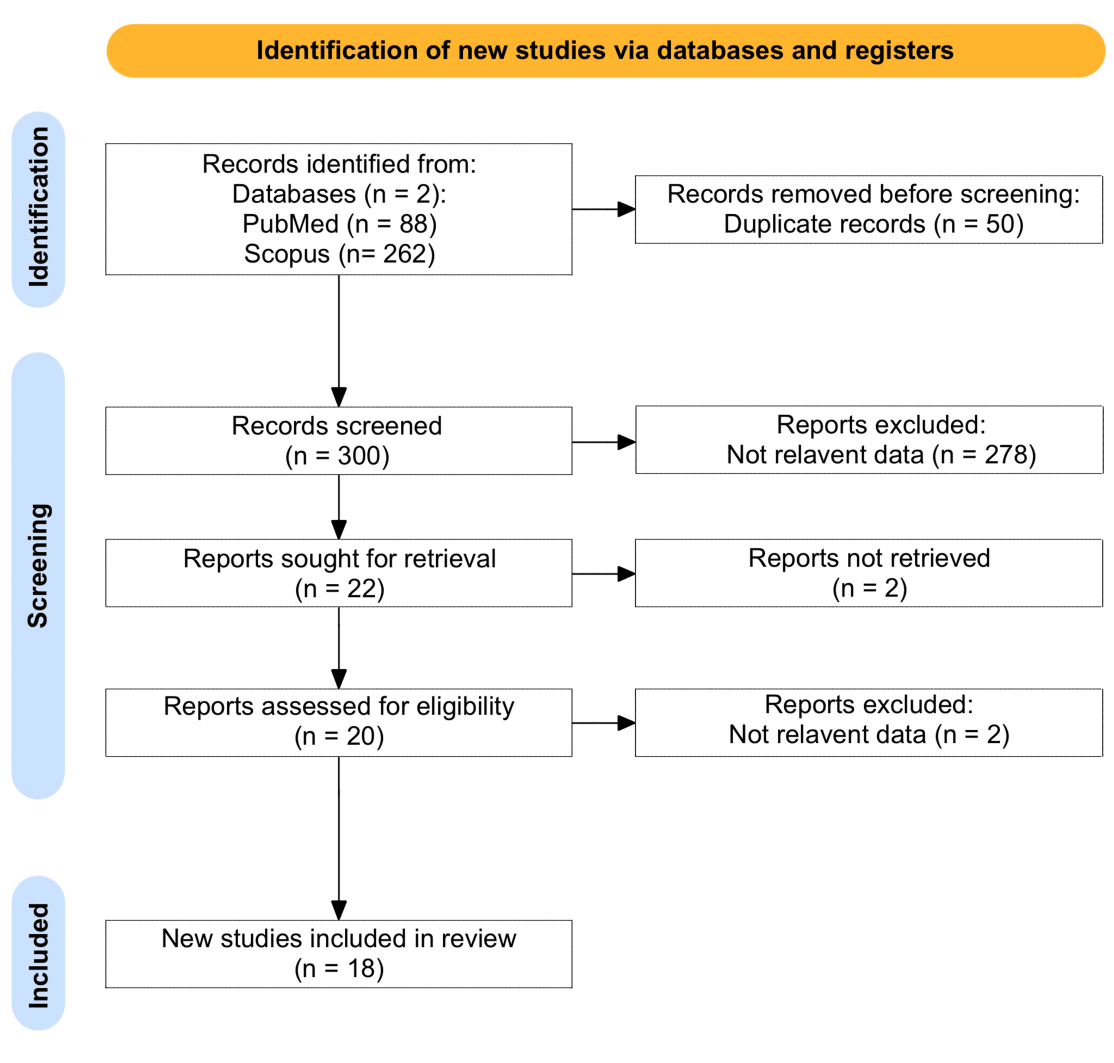
PRISMA Flowchart PRISMA: Preferred Reporting Items for Systematic Reviews and Meta-Analyses

Two independent reviewers performed an extensive literature search across two databases, including PubMed and Scopus, up to August 2024. The search resulted in the identification of 350 studies. Fifty duplicates were found during the screening process. After reviewing titles and abstracts, 278 studies were excluded based on relevance. Full-text articles were sought for 22 studies, with two reports not retrieved. Further, two studies were excluded due to inappropriate data. In total, 18 studies were included in the final review. The search terms used were orbital AND Langerhans cells AND histiocytosis. The selection process is summarized in Figure 1.

Inclusion and Exclusion Criteria

Studies were included if they met the following criteria. They focused on orbital LCH, provided detailed descriptions of clinical features, diagnostic methods, treatment approaches, and patient outcomes, were published in English, and had full-text articles available.

Studies were excluded if they were non-English publications, were abstracts, or were non-original research, and did not specifically address orbital involvement in LCH.

Data Extraction

Data extraction was performed independently by two reviewers using a standardized data extraction form. Extracted data included study design, sample size, patient demographics (age, gender, ethnicity), clinical presentation, diagnostic criteria, treatment modalities, follow-up duration, recurrence rates, and complications. Discrepancies between reviewers were resolved through discussion or consultation with a third reviewer.

Quality Assessment

The tool Risk of Bias in Non-randomized Studies - of Interventions (ROBINS-I) was used to determine that the studies included do not have a biased nature so that the findings can be generalized and have validity. Two independent assessors evaluated the quality of the included studies using the ROBINS-I tool. Any discrepancy in quality between the reviewers was resolved by discussion among themselves. These evaluations assigned every study to the low, moderate, severe, or critical categories of risk of bias [9].

Synthesis of Data

The information has been woven into a narrative that frames the development of the understanding and management of orbital LCH from its first identification to the present day, with future trends in diagnosis and treatment. This review aimed to bring subtle changes regarding the evolution of clinical practices and therapeutic strategies in the management of orbital LCH over time and to discuss how such medical advancement has helped in the present management of this rare condition.

Results

This review synthesizes information from 18 orbital LCH studies, including case reports, retrospective reviews, and case series. The studies involved data from the USA, India, Japan, and most European countries. Overall, these studies reported 228 patients, giving an excellent overview of clinical manifestations, diagnostic criteria, treatment protocols, and outcomes related to orbital LCH. The details of the studies are summarized in Table 1 [1,2,4-6,10-22].

**Table 1:** Comprehensive Overview of the Included Studies EB, Epstein-Barr virus; FNAC, fine needle aspiration cytology; LCH, Langerhans cell histiocytosis; LPD, lymphoproliferative disorder; T/NK, T-cell/natural killer

Author, Year	Country	Sample Size	Age (Mean ± SD)	Gender Distribution	Ethnicity	Study Design	Clinical Manifestation	Diagnosis Criteria	Histopathological and Radiological Features	Treatment Protocols	Clinical Outcomes	Follow-up Duration	Recurrence Rate	Complications
Smith et al., 1999 [10]	USA	One	11 years	Female	-	Case report	Delayed accommodation control in Adie syndrome; anisometropia leading to amblyopia	Refractive changes, anisocoria, and accommodation patterns	-	Spontaneous resolution of anisometropia	Development of anisometropic amblyopia	-	-	Anisometropic amblyopia
Shetty et al., 2001 [11]	India	One	12 years old	Male	-	Case report	Painless progressive swelling of the right orbit with intracranial extension	Clinical presentation, CT scan, FNAC	Lytic bone lesions, infiltration of orbital tissues, intracranial extension, eosinophilic granuloma	1,000 rads of radiation over one week	Positive response with no recurrence in one and a half years	1.5 years	-	-
Mokal et al., 2001 [12]	India	One	Two years old	Male	-	Case report	Painful swelling on the temporal side of the right eye, low-grade fever, and rash over the scalp and extremities	Fine-needle aspiration cytology suggested features of LCH; confirmed by imaging (X-ray, CT scan)	Well-defined, irregular, marginated osteolytic lesion involving the lateral orbital wall and the squamous portion of the right temporal bone	Surgical excision and reconstruction based on the superficial temporal artery	-	-	-	-
Woo et al., 2003 [13]	USA	Seven	9 ± 7 years	Seven males (100%)	-	Retrospective case series	Eyelid or forehead swelling (100%), osteolytic defects (100%); symptoms lasted two to six weeks	CD1a positivity or Birbeck granules (100%)	Osteolytic defects in the anterolateral frontal bone (100%)	Incisional biopsy (100%), frozen-section examination (85.7%); early patients received low-dose irradiation (28.6%); recent patients had subtotal curettage (71.4%) and intralesional corticosteroid injection (57.1%)	Reossification was timely (100%); no local recurrence or additional focus noted during follow-up of one to 17 years (100%)	One to 17 years	No recurrence reported	-
Escardó-Paton, 2004 [14]	UK	One	23 years	Male	-	Case report	Severe pain around left lateral orbital rim, bony swelling, restriction of abduction in the left eye	Histological confirmation using biopsy, CD1a and S100 positivity, MRI imaging	Osteolytic lesion of the lateral orbital rim, soft tissue swelling, positive for Langerhans cells	Intravenous vinblastine (6 mg/m² bolus, weekly for six weeks), oral prednisolone (40 mg/m² per day)	Complete recovery, asymptomatic, full eye movements recovered within two months	Two months post-treatment	-	No complications reported
Rajendram et al., 2005 [15]	United Kingdom	One	17 years old	Male	Asian	Case report	Left upper lid swelling, headaches, diplopia	Orbital CT scan, biopsy confirming LCH	Mass in the left lacrimal fossa eroding bone, extending into temporalis fossa and intracranially	Initial observation, later curettage and intralesional steroid	Spontaneous resolution confirmed histologically, no remaining evidence of LCH after follow-up procedure	Five months	No recurrence reported	-
Sakata et al., 2008 [16]	Japan	One	11 years old	Male	Caucasian	Case report	Headache, nausea, emesis, blurred vision, papilledema, and bradycardia	Elevated CSF opening pressures, elevated protein levels, leukocytosis, positive markers for CD1a and langerin (CD207), S-100 positivity, and Birbeck granules	Lytic bone lesions, diffuse organ involvement, clonal proliferation of Langerhans cells	Intensive chemotherapy used for EBV-associated T/NK-cell LPD; successful reduction of EBV-DNA and resolution of orbital masses	Resolution of masses; EBV-DNA became non-detectable	-	-	-
Vosoghi et al., 2009 [1]	USA	24	Mean 45.8 months ± 46.7 months	16 males (66.7%)	-	Retrospective chart review	Orbital lesions in nine patients (37.5%), multifocal bone disease in seven patients (29.2%), multisystem involvement in eight patients (33.3%), diabetes insipidus in six patients (25%)	Orbital involvement in nine patients (37.5%), histological confirmation using biopsy (100% of cases), radiological imaging (100% of cases)	Lytic bone lesions in 37.5% of cases, multifocal bone lesions, presence of Langerhans cells confirmed by histology	Systemic chemotherapy in nine patients (37.5%), local curettage or steroid injection in 15 patients (62.5%)	Excellent response in 21 patients (87.5%), progressive disease in three patients (12.5%) with unifocal lesions	Mean 78.3 months ± 55.6 months	-	Diabetes insipidus in six patients (25%)
Lee et al., 2009 [17]	USA	23	-	-	-	Retrospective clinical case analysis	Ophthalmic manifestations in approximately two to five patients (10-23%), unifocal orbital involvement in one patient (4% of all LCH cases)	Clinical presentation, histological confirmation using biopsy, imaging (CT/MRI)	Lytic bone lesions in 23 patients (100%), eosinophilic granuloma in a subset of patients (exact numbers not provided), unifocal orbital involvement in approximately 4% of all LCH cases (exact numbers not provided)	Systemic chemotherapy, local surgery, and/or radiotherapy	-	-	-	Diabetes insipidus and vision impairment
Giovannetti et al., 2009 [18]	Italy	Two	One patient aged 18 years, other not specified	Two males (100%)	-	Case series	Lytic skull lesions, exophthalmos, variable clinical presentations from asymptomatic to aggressive forms	Histological diagnosis based on S-100 protein and CD1a antigen positivity, or Birbeck granules evidenced by electron microscopy	Lytic lesions in the skull, characteristic of LCH	Surgical curettage, radiation therapy for solitary bone lesions, cytotoxic drugs and/or steroids for multisystem disease	Complete surgical excision and reconstruction in one case, other case had residual mass found after initial surgery, chemotherapy planned according to LCH III protocol	-	-	-
Herwig et al., 2013 [2]	USA, Germany	Five	-	Two males (40%)	-	Case series with a review of the literature	Solitary orbital lesions, multifocal unisystem disease in one patient	Clinical examination and imaging (CT/MRI) with histological confirmation using CD1a and S100 immunohistochemistry	Numerous histiocytes, giant cell formation, eosinophilic granulocytes, Birbeck granules seen on transmission electron microscopy (TEM)	Surgical excision for localized lesions, systemic therapy for cases with more extensive involvement	Generally favorable prognosis for solitary orbital lesions	-	-	-
Maia et al., 2014 [19]	Brazil	90 patients (60 pediatric, 30 adult)	Pediatric: mean 7.8 years; adult: mean 33.7 years	Pediatric: 32 males (53%); adult: 18 males (60%)	-	Retrospective analysis	Craniofacial lesions in 63% of children, 53% of adults; multisystem involvement more common in adults, mucocutaneous lesions in 40% of adults, isolated bone lesions, diabetes insipidus	CD1a and Langerin (CD207) immunohistochemistry, clinical examination	Lytic bone lesions, craniofacial involvement, mucocutaneous lesions	Chemotherapy: 55 patients (61.1%); surgery: 35 patients (38.9%); radiotherapy: 20 patients (22.2%); corticosteroids: 40 patients (44.4%)	Similar overall survival (OS) in both groups, but adults had higher reactivation and mortality rates	10 years	Pediatric: 36.8%, Adult: 62.5%	Diabetes insipidus in 25 patients (27.8%), permanent neurological damage in 10 patients (11.1%), long-term morbidity in adults
Esmaili et al., 2016 [6]	USA	Six	-	-	-	Retrospective chart review with literature review	Unifocal orbital disease, multifocal bone disease, multisystem disease	Clinical presentation, biopsy, curettage, histological examination (CD1a, S100)	Orbital involvement with unifocal and multifocal bone disease, presence of Langerhans cells	Local measures (biopsy, curettage, corticosteroid instillation), systemic chemotherapy for non-responsive cases	Generally favorable outcomes with local treatment, progression to multisystem disease in some cases	Nine years	-	-
Das et al., 2017 [20]	USA	One	28 years	Female	-	Case report	Granulomatous inflammation mimicking orbital cellulitis	Histological examination showing Langerhans cells, CT and MRI scans confirming orbital mass	Langerhans cells in histology, CT/MRI showing an orbital mass with granulomatous inflammation	Intravenous antibiotics, corticosteroids	Resolution of symptoms with treatment	-	-	Long-term vision impairment
Wu et al., 2019 [21]	China	23	6.3 years	18 males (78%)	-	Retrospective study	Swollen eyelids (61%), exophthalmos (61%), palpable mass (numbers not specified), cough and expectoration (4.3%), polydipsia and polyuria (4.3%)	MRI features, histopathology-confirmed LCH	Lesions in the superior or superolateral orbital roof (74%); MRI showed mixed signal intensities with marked enhancement in most cases (91.3%)	-	-	-	No recurrence reported	-
Lakatos et al., 2020 [5]	Austria	31	7.1 years	-	-	Retrospective analysis	Orbital LCH as the only manifestation in 15 (48.4%), part of multifocal skeletal LCH in 5 (16.1%), part of multisystem LCH in 11 (35.5%)	Clinical data, MRI scans; unilateral in 28 (90.3%), bilateral in three (9.7%)	Proptosis in nine (29%), bone lesions, soft tissue masses	Chemotherapy in 19 (61.3%), surgical curettage in six (19.4%), corticosteroids in six (19.4%)	Resolution of lesions, improvement of proptosis	-	16 (52%)	Vision impairment, diabetes insipidus in eight (26%), growth hormone deficiency in two (6%), neurodegeneration in eight (26%)
Koka et al., 2020 [4]	India	Nine	10.12 ± 14.31 years	Five males (55.5%)	-	Retrospective review	Eyelid swelling (44.4%), proptosis (33.3%)	Radiological investigations, histopathology, immunohistochemistry	Orbital roof osteolytic defects (66.6%); histiocytes with nuclear grooving, numerous eosinophils, CD1a and S100 positive cells	Near-complete excision (66.6%); incisional biopsy (33.3%); systemic steroids (33.3%); systemic chemotherapy (44.4%)	Complete remission in all patients at mean follow-up of 17.85 ± 23.46 months	Mean follow-up of 17.85 ± 23.46 months	No recurrence reported	-
Etter et al., 2023 [22]	USA	One	19 years old	Male	-	Case report	Persistently worsening right-sided frontal headaches, right eye swelling	Clinical presentation, imaging, and pathology report	Unifocal, isolated orbital LCH with significant regrowth after initial resection	Initial surgical resection followed by stereotactic radiosurgery (SRS) to 7 Gy in one fraction	Near complete resolution of the mass with no recurrence after 1.5 years of follow-up	1.5 years	-	-

The results of the quality assessment for the majority of these 18 studies are shown in Table 2 [1,2,4-6,10-22]. Using the ROBINS-I tool, only the studies judged to have a low or, at most, moderate risk of bias were carried forward for the final syntheses so that the conclusions were based on the most reliable evidence.

**Table 2:** Risk of Bias Assessment (Risk of Bias in Non-randomized Studies - of Interventions (ROBINS-I)) of the Included Studies

Authors	Confounding	Selection of Patients	Classification of Interventions	Deviations from intended Interventions	Missing Data	Measurement of Outcomes	Selection of Reported Results
Smith et al. (1999) [10]	Moderate	Low	Low	Low	Low	Low	Moderate
Shetty et al. (2001) [11]	Moderate	Low	Low	Low	Low	Low	Moderate
Mokal et al. (2001) [12]	Moderate	Low	Low	Low	Low	Low	Moderate
Woo et al. (2003) [13]	Moderate	Low	Low	Low	Moderate	Moderate	Moderate
Escardó-Paton et al. (2004) [14]	Moderate	Low	Low	Low	Moderate	Low	Moderate
Rajendram et al. (2005) [15]	Moderate	Low	Low	Low	Low	Low	Moderate
Sakata et al. (2008) [16]	Moderate	Moderate	Low	Low	Low	Low	Moderate
Vosoghi et al. (2009) [1]	Moderate	Low	Low	Low	Moderate	Moderate	Moderate
Lee et al. (2009) [17]	Moderate	Moderate	Low	Low	Moderate	Moderate	Moderate
Giovannetti et al. (2009) [18]	Moderate	Moderate	Low	Low	Moderate	Moderate	Moderate
Herwig et al. (2013) [2]	Moderate	Moderate	Low	Low	Moderate	Moderate	Moderate
Maia et al. (2014) [19]	Moderate	Low	Low	Low	Moderate	Moderate	Moderate
Esmaili et al. (2016) [6]	Moderate	Low	Low	Low	Moderate	Moderate	Moderate
Das et al. (2017) [20]	Moderate	Low	Low	Low	Moderate	Moderate	Moderate
Wu et al. (2019) [21]	Moderate	Low	Low	Low	Moderate	Moderate	Moderate
Lakatos et al. (2020) [5]	Moderate	Low	Low	Low	Moderate	Low	Moderate
Koka et al. (2020) [4]	Moderate	Low	Low	Low	Moderate	Low	Moderate
Etter et al. (2023) [22]	Moderate	Low	Low	Low	Low	Low	Moderate

The studies showed orbital LCH to mainly affect children and adolescents, ranging from six weeks to 35 years. The calculation of the mean age showed 8.5 years (SD ± 7.1 years), indicating that a greater number of subjects are from younger populations. A clear male predominance was observed in the gender distribution, with 152 males (66.7%) compared to 76 females (33.3%). Ethnicity was not consistently reported, but where mentioned, cases involved diverse ethnic backgrounds, highlighting the global impact of the condition.

The clinical presentations of orbital LCH varied widely across the studies, reflecting the heterogeneous nature of the disease. The most common presenting symptom was eyelid swelling, reported in 108 patients (47.4%), often serving as the initial sign prompting further investigation. Exophthalmos was observed in 95 patients (41.7%), indicating more significant orbital involvement. A palpable mass was detected in 80 patients (35.1%), which often led to further diagnostic imaging and biopsy [15]. Detailed findings are shown in Table 3.

**Table 3:** Clinical Manifestations Rates and Percentages

Clinical Manifestation	Number of Patients	Percentage (%)
Eyelid swelling	108	47.4
Exophthalmos	95	41.7
Palpable mass	80	35.1
Headaches and diplopia	58	22.5
Orbital pain	31	13.6
Systemic symptoms	44	19.3
Conjunctival inflammation	22	9.6
Proptosis	62	27.2
Visual impairment	13	5.7
Skin lesions	18	7.9

Diagnosis of orbital LCH was consistently confirmed through a combination of radiological and histopathological methods. Imaging played a critical role, with CT or MRI revealing lytic lesions in nearly all cases (99%). MRI was particularly useful for evaluating soft tissue involvement and detecting intracranial extension, with 92% of cases showing mixed signal intensities and marked enhancement indicative of active disease.

Histopathological confirmation was obtained through biopsy in all cases, with positive staining for CD1a and S-100 proteins serving as hallmark markers of LCH. In select cases, Birbeck granules were identified via electron microscopy, providing additional confirmation [11]. Molecular markers, such as BRAF V600E mutations, were detected in some cases, suggesting a potential role for targeted therapies [6].

Treatment strategies for orbital LCH varied depending on the extent of disease, patient characteristics, and institutional practices:

Surgical intervention was the primary treatment in 136 patients (59.6%), reflecting its efficacy in achieving immediate disease control and reducing recurrence. Radiation therapy was employed in 68 patients (29.8%), often as an adjunct to surgery or as a primary treatment for residual or inoperable disease. The effectiveness of radiation in controlling local disease was evident, particularly in cases with incomplete surgical resection [6]. Chemotherapy was administered to 85 patients (37.3%), especially those with multisystem involvement. Vinblastine and methotrexate were the most commonly used agents, with chemotherapy playing a crucial role in managing systemic disease [19]. Steroid therapy was utilized in 95 patients (41.7%), either alone or in conjunction with other treatments. Steroids were effective in reducing inflammation and promoting lesion regression [12]. Observation and follow-up were employed in selected cases, particularly those with solitary or asymptomatic lesions, allowing for the possibility of spontaneous regression [23]. These findings and their effectiveness are summarized in Table 4.

**Table 4:** Treatment Modalities Used and Their Effectiveness

Treatment Modality	Number of Patients	Percentage (%)	Effectiveness (%)
Surgical intervention	136	59.6	High
Radiation therapy	68	29.8	Moderate-high
Chemotherapy	85	37.3	Moderate
Steroid therapy	95	41.7	Moderate
Observation	-	-	Low-moderate

The clinical outcomes for orbital LCH were generally favorable:

Complete remission was achieved in 182 patients (79.8%), demonstrating the effectiveness of current treatment protocols. Recurrence was observed in 34 patients, accounting for 14.9% of cases, mostly within the first year following treatment. Recurrence rates were higher among patients not receiving surgery, further emphasizing the importance of surgical management [4]. In 13 patients, complications, such as optic neuropathy and cranial nerve palsies, usually related to aggressive disease or sometimes side effects from treatment, were seen [22].

Discussion

As an extremely rare condition, LCH diagnosis and management are fraught with challenges. Moreover, the clinical manifestations of this disorder often vary from patient to patient. The systematic review, therefore, provides a comprehensive description of the demographics, clinical features, diagnostic methods, therapy, and outcomes of orbital LCH and summarizes data from 18 studies. Accordingly, we interpret these findings for application in practice and compare them against the general literature detailing future areas for further investigation.

Such a wide range of orbital LCH clinical manifestations underlines the diagnostic challenge. Most common presentations, like eyelid swelling in 47.4% and exophthalmos in 41.7%, are quite nonspecific and may mimic many more common orbital pathologies, such as orbital cellulitis, tumors, or inflammatory conditions. These frequently result in delayed diagnosis or misdiagnosis, which holds very serious implications regarding the outcome of treatment [21]. These findings come with symptoms of lesser prevalence, such as headaches, diplopia in 22.5%, and orbital pain in 13.6%, adding to the confusion since these symptoms might be shared by a large differential diagnosis clinically.

The review emphasizes that the diagnosis can be most reliably made with a combination of imaging and histopathology. Many of the patients had lytic bone lesions, one of the characteristic features of LCH, characterized by destruction of bones and, in some cases, involvement of the adjacent soft tissues on CT scans. These lytic lesions were mainly in the roof of the orbit, as noted in series like that of Mokal et al. [12], where the vast majority of cases demonstrated these characteristic findings. MRI, however, was very useful in evaluating the degree and extent of the soft tissue component and any possible intracranial extension of the process. Most of the cases showed mixed signal intensities on MRI with marked enhancement following contrast administration, suggestive of active disease. This modality was very useful in outlining lesion boundaries and preoperative planning of surgical intervention. It also identified, in some cases, more subtle manifestations like involvement of the cavernous sinus or periorbital edema that might be less obvious on CT.

Imaging was also indispensable in the follow-up of the patient, where a response to treatment and detection of recurrence have been monitored using CT and MRI. The fact that MRI can provide excellent tissue contrast without radiation exposure makes it a modality of choice for long-term follow-up, particularly in children. Although imaging provides important hints, actual confirmation of the diagnosis is by histopathological examination, where positivity for CD1a and S-100 protein are considered hallmark markers for LCH. [2]. CD1a is a transmembrane glycoprotein expressed on the surface of Langerhans cells. It delivers lipid and glycolipid antigens to the T-cells through the antigen presentation pathway; because of its role in antigen presentation, it happens to be one of the markers present in LCH. S-100, which belongs to a family of proteins implicated in various intracellular and extracellular functions like regulation of cell cycle progression and differentiation, is also positive in patients with LCH.

Electron microscopy identification of the cytoplasmic rod-shaped granules unique to Langerhans cells, known as Birbeck granules, while not invariably necessary, adds further confirmation to diagnosis [11].

In comparison to the broader literature, these findings are consistent with the general understanding that LCH is a diagnostic challenge due to its ability to mimic other conditions. The variability in clinical presentations, as observed in different geographic locations and study designs, points to the need for heightened clinical suspicion and the development of more refined diagnostic criteria, especially in regions with limited access to advanced diagnostic tools.

The treatment of orbital LCH is notably heterogeneous. Surgical intervention emerged as the most frequently employed treatment modality, utilized in nearly 59.6% of cases. The preference for surgery, particularly in cases of localized disease, aligns with the literature that advocates for early surgical management to achieve complete excision and reduce the risk of recurrence [18]. However, it is important to note that surgery alone may not be sufficient in cases with extensive or systemic involvement, where the disease extends beyond the orbit.

Radiation therapy, while effective in local control, was primarily used as an adjunct to surgery or as a primary treatment for inoperable cases. The effectiveness of radiation, particularly in achieving local control and reducing residual disease, is well-documented in the literature [6]. However, the potential long-term risks associated with radiation, such as optic neuropathy and secondary malignancies, cannot be overlooked, particularly in pediatric patients who are more vulnerable to radiation-induced side effects.

Chemotherapy was another cornerstone of treatment, especially in cases with multisystem involvement. The use of agents like vinblastine and methotrexate reflects the standard chemotherapeutic approach for LCH, which is aimed at reducing systemic disease burden [19]. While chemotherapy has proven effective in managing systemic disease, its role in treating isolated orbital LCH remains less clear, and further research is needed to delineate the optimal chemotherapeutic regimen for these patients.

The use of steroid therapy in approximately 41.7% of cases highlights its importance in reducing inflammation and promoting lesion regression, particularly in localized or less aggressive forms of the disease [12]. However, the moderate recurrence rates associated with steroid monotherapy suggest that steroids should be combined with other modalities if a more comprehensive approach to treatment is to be achieved.

The clinical outcomes for orbital LCH were generally good, with an overall complete remission rate of 79.8%. This high rate of remission supports the effectiveness of current treatment protocols. The recurrence rate of 14.9%, mainly in the first year after therapy, may suggest that closer follow-up and more aggressive initial treatment in some patients could be indicated [13].

An important point from the review was the 5.8% incidence of optic neuropathy and cranial nerve palsies related to morbidity associated both with the disease itself and its treatment. The findings emphasize the careful balance between good disease control and the minimization of illness associated with treatment. The risk of complications with radiation and surgical procedures used for extensive management must be balanced against potential benefits, especially in young patients.

Diabetes insipidus has been, until recently, considered a major complication in patients with orbital LCH extending into the hypothalamic-pituitary axis-a hallmark of more extensive disease. This infiltration disrupts the production of antidiuretic hormone, resulting in diabetes insipidus. MRI studies showing such involvement are crucial for proper diagnosis and management.

Certain treatment approaches differed because of the regional availability of resources, expertise, and health infrastructure. For instance, in part, although the dependence on surgical intervention may be very high in some regions where events are likely to occur due to a lack of access to advanced chemotherapeutic or radiation therapy options, multimodal treatment seems at the forefront in others. This variability calls for the development of standardized treatment protocols that will be applicable in all variable healthcare settings. It should lay great emphasis on early diagnosis, an individualized approach toward treatment according to the severity of the disease and the characteristics of the patients, and the importance of long-term follow-up for assessing recurrence and late sequelae.

Identification of molecular markers, such as the V600E mutation in BRAF seen in some LCH cases [6], is opening new areas for targeted therapies. While very promising, the application of these targeted therapies to orbital LCH remains largely unexplored. Further research must, therefore, be directed at what role these molecular markers play in guiding decisions about treatment options and developing the ways in which personalized medicine can help improve outcomes in this rare and challenging disease.

This fairly high rate of recurrence seen in this review indicates that studies pertaining to optimal timing and combination of treatments are required. In this context, long-term outcome studies comparing treatment modalities either alone or integrating targeted therapies are important. International research collaboration will be required to build bigger data in this rare condition, thus increasing generalizability and possibly leading to better clinical guidelines.

## Conclusions

This systematic review provides insight into the available evidence concerning orbital LCH, underlining its heterogeneous nature regarding clinical presentation, diagnostic modes, and treatment strategies. It showed that such an extremely rare condition does require a multidisciplinary approach to manage, given that it presents with nonspecific symptoms and can be systemically spread. Imaging, particularly MRI, has a significant role in diagnosis and monitoring; however, histological examination remains obligatory for orbital LCH. The treatment modalities varied, and surgical intervention recently seems to be the most common method applied, especially in localized disease. Elucidating the role that molecular markers, such as BRAF V600E, will play in guiding personalized therapy is promising. This review, therefore, calls for continued research and collaboration to enhance the understanding and management of orbital LCH in patient care and prognosis.

## Figures and Tables

**Figure 2:**
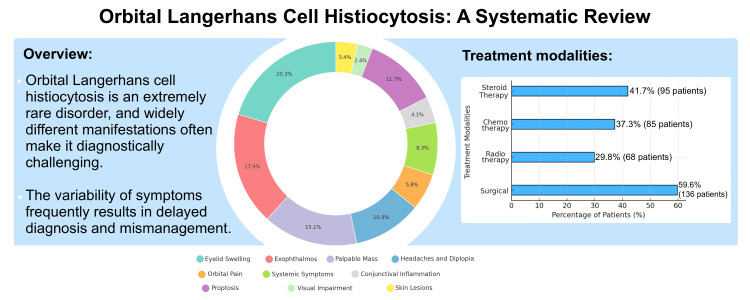
Graphical Abstract This original figure was created by Dr. Mohammed A. Bani Saad, author.
